# Validity of Activity-Based Checks (ABCs) of Pain, A Functional Pain Scale

**DOI:** 10.1016/j.pmn.2025.09.010

**Published:** 2025-10-09

**Authors:** Celina G. Virgen, Robert Wright, Bryan Renslo, Tuleen Sawaf, Hanna Moradi, Maria Edelen, Jennifer Villwock

**Affiliations:** * Department of Otolaryngology- Head and Neck Surgery, University of Kansas Medical Center, Kansas City, KS; † School of Medicine, University of Kansas, Kansas City, KS; ‡ The Brigham and Women’s Hospital, Harvard Medical School, Boston, MA

**Keywords:** Chronic pain, Pain scale, Validation, Functional pain, ABCs

## Abstract

**Purpose:**

The Activity-Based Checks of Pain (ABCs) is a pain assessment tool incorporating activities of daily living and instrumental activities of daily living. This instrument is designed to focus on functional capabilities and limitations due to pain. This study was designed to validate the factorial structure of the ABCs and assess its use in participants with chronic pain.

**Methods:**

Participants were recruited in two phases. Phase one optimized the design of the ABCs, with 297 subjects selecting their preferred icon for each function and rating its understandability. The most preferred and understandable icons were then used in phase two, where 304 participants with chronic pain completed the ABCs, the Patient-Reported Outcomes Measurement Information System (PROMIS) with additional PROMIS items that were analogous to the ABCs functions but not represented in the PROMIS-29, and the Brief Pain Inventory (BPI). Data were analyzed using exploratory factor analysis and confirmatory factor analysis.

**Results:**

Four factor loadings resulted in: multiplanar activities, sitting and/or hip flexor pain, walking and/or ambulation, and pain interference with lightweight unilateral activities. High internal consistency was demonstrated for all four factor loadings (0.623-0.879, 0.577-0.824, 0.512-0.841, 0.519-0.817, respectively). Correlations between items in the ABCs, PROMIS, and BPI resulted in moderate to strong correlations. Test-retest reliability was moderate to strong (intraclass correlation coefficient: 0.74).

**Conclusions:**

The results confirm the ABCs as a valid and reliable tool for assessing the impact of pain on function in patients with chronic pain.

## Introduction

Pain is a unique experience that is personal, private, subjective, and can become a barrier to achieving an enjoyable quality of life (Breivik et al., 2008). Assessing pain can provide insight into the type of pain experienced by patients, if current treatment is adequate for pain control, or if additional interventions are merited (Gordon, 2015). Despite the availability of several pain assessment tools, an inherent disconnect remains between patient-clinician communication regarding pain intensity and management goals (Henry et al., 2017). Patients have expressed preference for and trust in clinicians who go beyond focusing on physical pain (Haverfield et al., 2018). The impact of pain is multidimensional, affecting quality of life, social interactions, mental health, and activities of daily life (Langley et al., 2011). A patient-centered functional approach may allow for meaningful and feasible improvements in quality of life (Mallick-Searle et al., 2021).

Current pain assessment tools limit the clinician in fully evaluating the patient’s pain experience. Popular tools such as the Numeric Rating Scale (NRS) and Wong-Baker Faces Pain Rating Scale (FACES) are highly subjective, which may misrepresent pain intensity score and tolerable pain while overshadowing function and patient progress (Adeboye et al., 2021; Van Dijk et al., 2012). A patient responding on a 0 to 10 scale may express 10/10 pain but still be able to perform activities of daily living (ADLs), while another patient also responds 10/10 and is unable to go for a short walk because of such pain. Error may be introduced when attempting to gauge the intensity of pain at different points in time, during activities, and movements. Other tools that do incorporate physical function include the Brief Pain Inventory (BPI) and the Patient-Reported Outcomes Measurement Information System (PROMIS). The BPI is meant to provide insight into the severity of pain experienced and the effect pain has on daily functioning in multiple domains (Atkinson et al., 2011). The PROMIS measures various health aspects, including self-efficacy, fatigue, anxiety, depression, pain interference (the extent to which pain affects performing activities and enjoying life), sleep, and physical function (Amtmann et al., 2010; Cella et al., 2007). Unlike these tools, the Activity-Based Checks of Pain – Functional Pain Scale (ABCs) is a visual info-graphic pain assessment tool. Simple visual icons representing various ADLs—activities for basic survival and/or living—and instrumental activities of daily living (IADLs)—more complex activities for independent living—were designed to reduce the amount of cognitive effort to interpret information by those completing the survey (Edemekong et al., 2024; Guo & Sapra, 2024).

The ABCs is meant not only to evaluate pain but also to understand how pain interferes with and limits activities. Pilot studies were initially created prior to final development and validation, utilizing common postrecovery functions after a surgical procedure intended to compare the scale against the NRS and report the need for pain medication (Hierl et al., 2021; Ho et al., 2022; Warnky et al., 2023). Results demonstrated comparable outcomes to the NRS; however, at the time of these studies, the scale consisted of fewer ADL and IADL icons, all of which were selected by the authors. The current ABCs scale is more comprehensive than the initially piloted scale and is intended for broad use. It also involved participants in the design, who selected icons for ADLs, IADLs, and bidirectional arrows, as described in the methods. The final development and effectiveness of the ABCs have not been previously validated against other functional pain scales. The objectives of this study are to (1) investigate and validate the factorial structure of the ABCs and (2) evaluate its use in chronic pain patients.

## Methods

Institutional Review Board and ethical approval for this study were obtained from the University of Kansas Medical Center, study 00142379. This study was previously posted on the medRxiv preprint server on January 29, 2024 (Virgen et al., 2024).

### Target Population and Recruitment

Participants were identified and recruited through Prolific, an online source used to identify volunteer research participants based on study criteria (Prolific, 2014). Prolific reports that participants are vetted extensively to detect bots and remove participants considered low- to no-effort responders. Data is constantly monitored internally to ensure high-quality results. Compensation is provided to participants for participation in the surveys. All participants recruited completed electronic consent via Prolific prior to participating in the study. All surveys were completed electronically. Participant inclusion criteria consisted of age 18 years or older, residing in the United States, and English as the primary language. Participants were excluded if they did not meet these criteria. Two separate cohorts were recruited. The first cohort was used to determine the best-fit icons for each activity or function, described in terms of icon comprehensibility and strength of association. The best-fit icons were incorporated into the next iteration of the ABCs. The arrow style used to indicate the pain level on the pain spectrum was also tested, with participants selecting between a black arrow, a colorful arrow, and arrows annotated with descriptors of mild, moderate, or severe pain. Field testing of the icons within the ABCs was done in the second cohort. The experience of chronic pain for at least 6 months was an additional inclusion criterion for this cohort. The test-retest cohort retained the same inclusion criteria as the second cohort, given that these were the same participants.

### Content Validity: Cohort 1

Participants were presented with the name of the function or activity of interest (e.g., preparing a meal) and four icons meant to represent it. They selected their preferred icon based on relevance to the function (In your opinion, which of these images represents preparing a meal?), comprehensibility (Is it easy or difficult to understand that these images represent preparing a meal?), and strength of association (Which of the images best represents the ability to prepare a meal?). The most-selected and best-fit icon was then used in the ABCs presented to cohort two (chronic pain participants).

### ABCs of Pain Functional Scale

**Figure 1.**
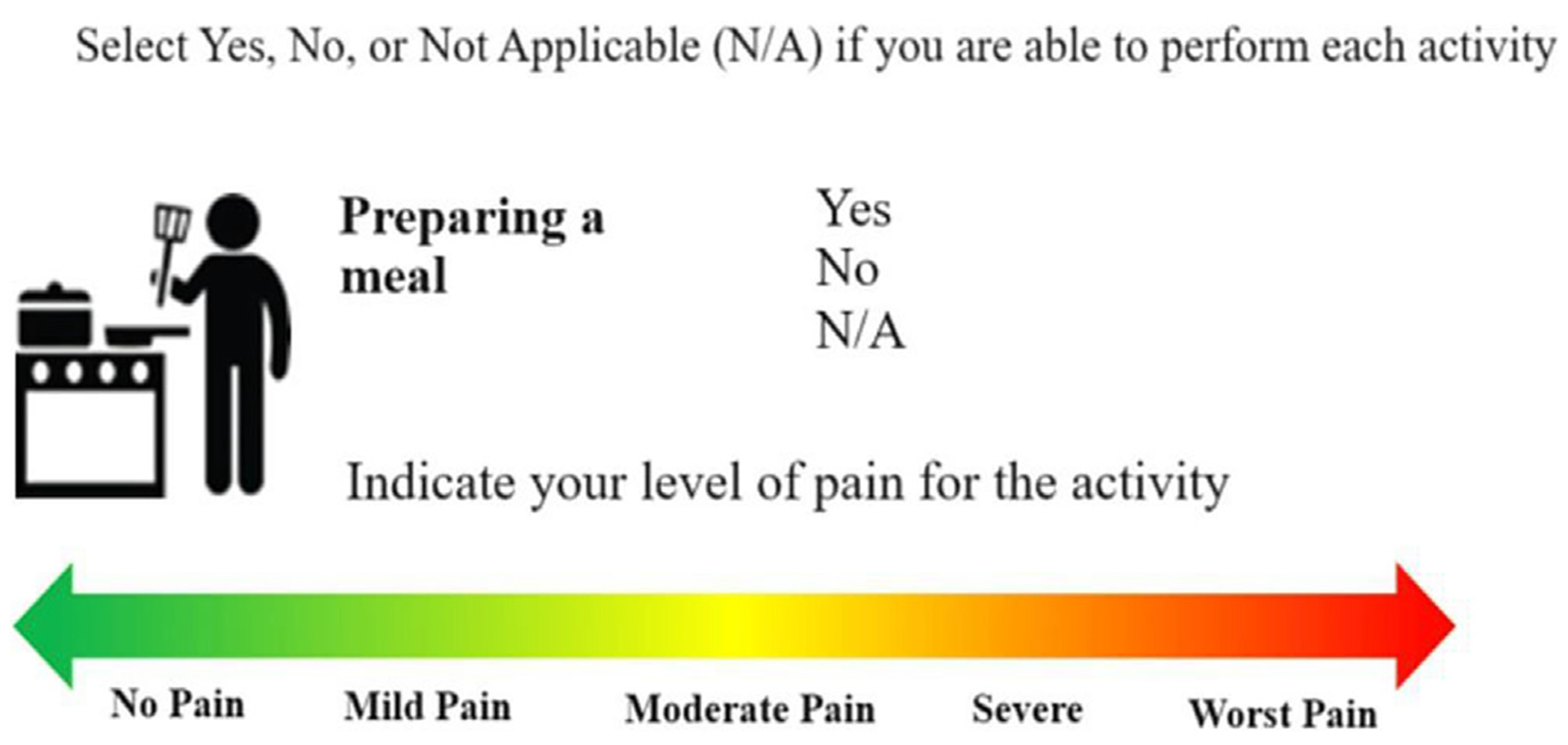
Activity-Based Checks of Pain (ABCs) activity example.

Initial development of the ABCs included selection of icons representative of the activities or functions: walking, jogging and/or running, prolonged standing, prolonged sitting, sleeping, getting out of bed, sitting up, getting out of a chair, doing laundry, grocery shopping, walking a dog, playing with a child, bathing, housework, picking up 20 pounds, preparing a meal, eating, opening a bottle to take medicine, walking upstairs, talking on the phone, getting dressed, personal care and/or grooming, driving, and toileting. Completion of the ABCs was done in a stepwise form. First, participants were asked if they were able to perform the given activity and to select, “yes,” “no,” or “not applicable” as an option (Fig. 1). If the participant responded “yes” to such activity, then they were able to indicate their level of pain on a bidirectional arrow from “no pain” to “worst pain.” If they selected “no,” a follow-up question asks why. Participants may select that the activity is too painful or for other reasons. If “too painful,” then participants were able to indicate on the bidirectional arrow how much pain they thought they would be in if the activity was attempted. The bidirectional arrow was scored with 0 being “no pain” to 10 being the “worst pain.” The distribution of the ABCs to participants was done electronically, as the goal is for the ABCs to be available in electronic format.

### Construct Validity: Cohort 2

Participants were recruited to evaluate the construct validity of the ABCs against the BPI and the PROMIS, both of which are previously validated measures of pain. For the BPI, higher scores indicate worse pain and pain interference (Atkinson et al., 2011). Items in the BPI regarding everyday pain and percentage of pain relief with treatment are optional and are not included in the scoring. A score of 1-4 is considered overall mild pain, 5-6 is moderate pain, while an average score of 7-10 is severe pain. The PROMIS responses are scored on a 5-point Likert scale, ranging from 1 to 5 (Cella et al., 2007). The PROMIS also includes a numeric rating scale that is scored from 0 to 10. A total score is based on the sum of all items, producing a score between 4 and 20, which is then converted into a T-score with a mean of 50 and standard deviation of 10. The ABCs contained more functional items than the PROMIS. As such, the PROMIS item bank was searched to include items analogous to all the ABCs functions, allowing comparisons between ABCs and PROMIS items to be made. Twenty-three items were added related to pain inference and physical function, making the PROMIS a total of 52 questions. The times necessary to complete the BPI, PROMIS, and ABCs were also recorded. To assess test-retest reliability, cohort two participants were invited to complete pain assessments 2 weeks after their initial participation.

### Statistical Analysis

All study data were collected and managed using REDCap electronic data capture tools and then exported to R (version 4.2.1) for analysis (Harris et al., 2009, 2019).

Descriptive data, categorical variables, and continuous variables are reported as counts, percentages, and means with standard deviations. To evaluate construct validity, an exploratory factor analysis (EFA) was conducted. The factor analysis results were used to determine the number of latent factors and their relationship to theory. A maximum likelihood analysis was conducted with oblique rotation. Confirmatory factor analysis (CFA) confirmed the factor structure through EFA. Model fit was assessed using the chi-square test, comparative fit index (CFI), Tucker-Lewis index (TLI), and root mean-square error of approximation (RMSEA). Modification indices identified areas of misfit and refined the model.

Internal consistency was assessed using Cronbach’s alpha coefficient for each factor. A coefficient alpha of >0.70 was considered acceptable. Intraclass correlation coefficient (ICC) was used to evaluate test-retest reliability, with the minimum acceptable value set at >0.70 (Koo & Li, 2016).

Convergent validity was assessed by examining the correlation between the new pain scale and other measures of pain interference. Pearson’s correlation coefficients between the ABCs, BPI, and PROMIS were calculated. The strength of correlation was interpreted as follows: r <0.3 = weak correlation, r = 0.3–0.6 = moderate correlation, and r >0.7 = strong correlation (Akoglu, 2018).

## Results

### Participant Demographics

**Figure 2.**
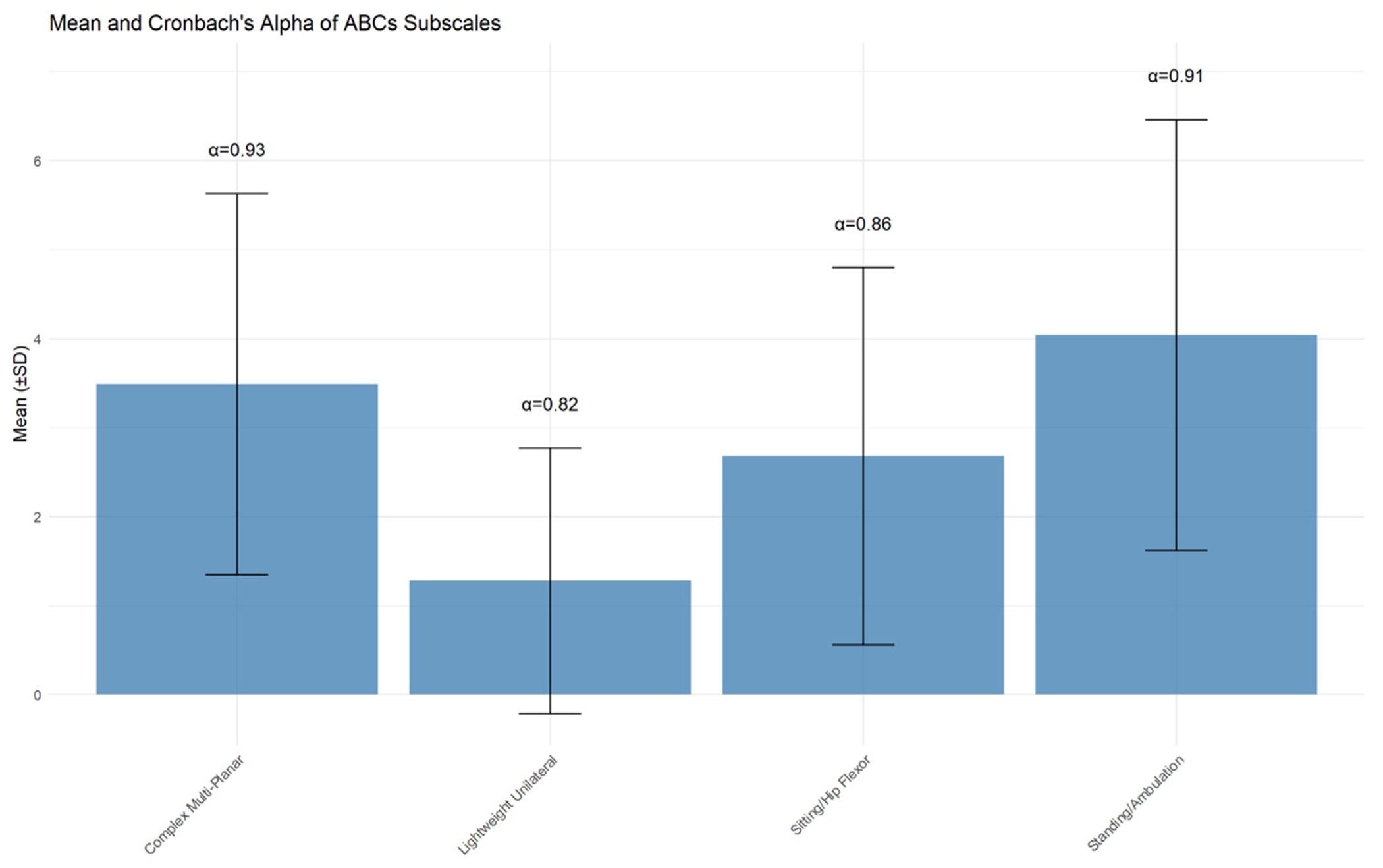
Score distributions, response frequencies, and averages for the ABCs. ABCs = Activity-Based Checks of Pain.

There were 297 participants in cohort 1 and 304 in cohort 2 (Table 1). In cohort 1, 55% were female, with 70.1% identifying as white and a median age of 34. Participants in cohort 2 were 51% male and 80% white, with a median age of 38 years, and over half reported chronic pain issues of at least 5 years or more. Age was demonstrated to be of non-normality in all cohorts. Notably, the average times to complete the BPI, PROMIS-29, and ABCs were 2.4 minutes, 3.8 minutes, and 3.9 minutes, respectively. The score distribution and pattern response for the ABCs of each activity are reported in Fig. 2.

### Factor Structure and Analysis

**Figure 3.**
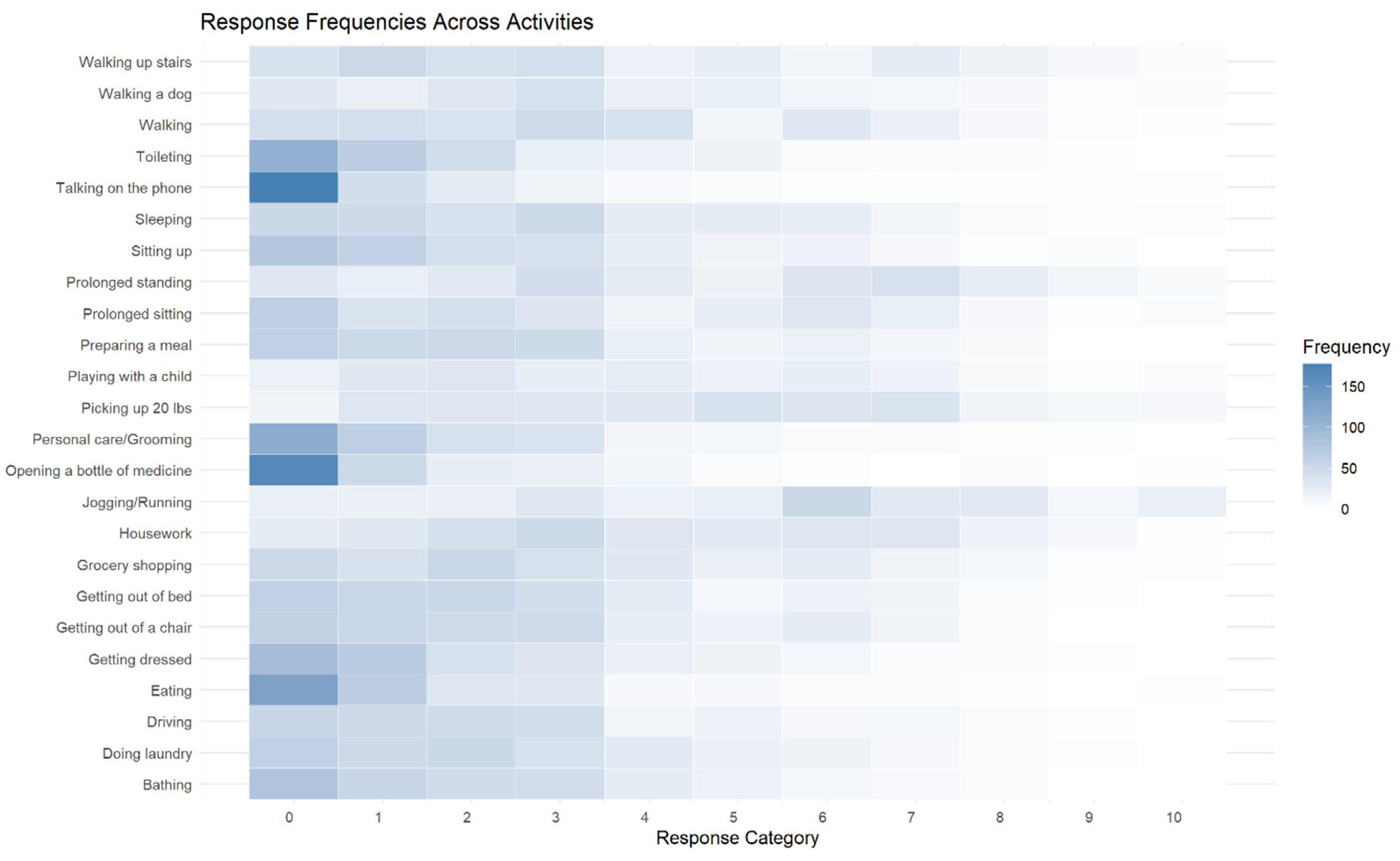
Maximum likelihood factor bases loadings for the retained 18 items of the ABCs. ABCs = Activity-Based Checks of Pain.

The factor analysis revealed a four-factor solution (Fig. 3). The first factor consisted of six items and had loadings of 0.623 to 0.879 for items 1 to 6, with 24% variance. The second factor consisted of four items and had loadings of 0.577 to 0.824 for items 6 to 10, with 17% variance. The third factor comprised four items and had loadings of 0.512 to 0.841 for items 11 to 14, with 16% variance. The fourth factor had four items and loadings of 0.519 to 0.817 for items 15 to 18, with 13% variance. The factor loadings suggest the factors measure pain interference with (1) complex multiplanar activities, (2) sitting and/or hip flexor, (3) walking and/or ambulation, and (4) lightweight unilateral activities.

The results of the CFA supported the four-factor structure (Table 2; *χ*^2^ = 318.8, df = 129, *p* < .001). The CFI was 0.93, the TLI was 0.91, and the RMSEA was 0.08, all of which indicate a moderate fit.

### Reliability

Standing and/or ambulation, as measured by both Cronbach’s alpha and McDonald’s omega, demonstrated high reliability with values of 0.937 and 0.941, respectively (Table 3). Similar patterns of high internal consistency were observed for the remaining factors. These results suggest all four factors possess robust internal consistency. The congruence of the alpha and omega values for each factor reinforces the reliability of the measurement model. Participants who initially completed the survey were invited to participate in the collection of test-retest data 2 weeks after initial responses. Test-retest reliability was assessed using the ICC. The ICC total scale score was 0.74, indicating moderate test-retest reliability.

### Convergent Validity

Convergent validity was assessed by examining the correlation between the scales (Table 4). Correlations between analogous items from pain scales are shown in Table 5. All correlations were found to be moderate to strong.

## Discussion

The aim of this study was to investigate and validate the factorial structure of the ABC—a novel functional pain scale that utilizes a visual icon-based format and assesses pain in the context of its impact on the ability to perform activities of interest—and evaluate its use in chronic pain patients. The results indicate the scale functions well in measuring pain interference. This multidimensional scale measures pain interference with (1) complex multiplanar activities, (2) sitting and/or hip flexor pain, (3) walking and/or ambulation, and (4) lightweight unilateral activities. Test-retest reliability was moderate to strong, with an ICC of 0.74. Fit indices from the CFA support construct validity, and Cronbach’s alpha supports the internal consistency of the ABCs. The results confirm the ABCs as a valid and reliable tool for assessing pain’s impact on function in chronic pain patients.

CFA with a four-factor structure supported the validity of the ABCs. Among the four factors, similarities were noted in the constructs of complex multiplanar activities, sitting and/or hip flexor pain, walking and/or ambulation, and lightweight unilateral activities. There were cross-loadings for items such as “walking a dog” with the walking and/or ambulation group of items. While this intuitively makes sense, the item remained within the multiplanar subscale since walking a dog is an activity that involves more than simply walking. Similarly, housework, getting out of bed, eating, and personal care and/or grooming show similar signs of cross-loading onto factors that incorporate some of the aspects of the activity.

In the current study, the ABCs were found to correlate with the BPI and PROMIS scales, demonstrating high convergent validity. Previous studies comparing results between the BPI and PROMIS for assessing pain have found variable results. Kean et al. evaluated persistent musculoskeletal pain in 250 patients before and after interventions using the BPI, PROMIS pain interference measures, the 2-item Bodily Pain subscale of the Medical Outcomes Study, 36-Item Short Form Survey bodily pain subscale, and the 3-item Pain Intensity, Enjoyment of Life, General Activity (PEG) Scale (Kean et al., 2016). Compared with the BPI, the PROMIS was less sensitive in re-evaluating pain and detecting changes or improvements after intervention. In contrast, Chen et al. found no significant difference in 759 patients between the BPI and PROMIS in being able to assess pain at baseline and at 3-6-month follow-up (Chen et al., 2019). Variability in responses to pain assessment can be highly context-specific; for example, the complex nature of pain, pain location, personal perceptions of pain, timing of pain evaluations, which may explain both consistency in results and lack thereof, depending on study design (Breivik et al., 2008; Khanom et al., 2020). The ABCs were able to capture tolerable and intolerable activities as reported by participants while demonstrating moderately strong test-retest reliability. The ability of the ABCs to assess pain with activities may add value for the pain management team, who are trying to assess specific functional outcomes related to pain management interventions.

The ABCs employs a straightforward 0-10 pain scale as a heuristic, making it easily comprehensible and applicable for both pain management nurses and patients. It builds on this concept with subscales created from items grouped based on similar movements and body parts, thus allowing for more nuanced evaluation of pain. Capturing this more nuanced data can help facilitate better communication of pain and functional goals. Tracking and understanding pain with specific activities can help with understanding and improving pain treatments and interventions.

When comparing time to completion among the three scales, the ABCs’ completion time was similar to the PROMIS items: 3.9 minutes versus 3.8 minutes, respectively. However, compared with the BPI (2.4 minutes), the ABCs took approximately 1.5 minutes longer to complete. The future intent for the ABCs is to create an adaptable scale that is personalized to each patient’s functional priorities. Not every activity or function in the current ABCs scale applies to all patients. The PROMIS has become a well-recognized scale with numerous bank items that can be added or removed from the scale based on clinician preference and purpose for its use. Times to completion for adaptations of the PROMIS have been previously reported to range from 3.3 minutes to 6.4 minutes (Kadri et al., 2018; Porter et al., 2021). In comparison, the BPI has been reported to take up to 5 minutes (Hassett et al., 2020). Time to completion for the BPI and PROMIS varies depending on the target population and the version of the scales being implemented. Future adaptations of the ABCs, with the ability to select pertinent activities, may further decrease time to completion.

### Limitations

Although the study’s results indicate the validity of the ABCs, it was not without limitations. First, participants were recruited through a survey distribution site based on self-reported chronic pain. Previous studies have demonstrated that Prolific provides higher quality responses than directly surveying participants (Douglas et al., 2023; Peer et al., 2022). Based on such findings, the research team does not believe this to have been a significant limitation. Second, test-retest reliability was assessed, given the complexity of pain and individual lifestyles, levels of pain, and location may fluctuate. Despite this, the test-retest results do not appear to have been affected. The team acknowledges that there is variability within the chronic pain patient population. Chronic pain from different etiologies may manifest differently. However, this is not believed to have significantly influenced the results, particularly when making comparisons between assessments, as each individual served as their own control. While the four subscales cover a broad range of activities, there are certainly more activities that could be included in the ABCs depending on the priorities of a particular patient population or recovery following certain procedures. Additional studies will address such patient- and procedure-specific priorities and outcomes.

## Conclusion

This study successfully validated the ABCs, a novel functional pain scale that employs a visual icon-based format to assess pain interference in patients with chronic pain. The scale’s multidimensional structure, as identified through EFA and confirmed through CFA, measures pain interference in four distinct areas: complex multiplanar activities, sitting and/or hip flexor pain, walking and/or ambulation, and lightweight unilateral activities. The ABCs demonstrated moderate to strong test-retest reliability, and fit indices from the CFA supported its construct validity and internal consistency. The scale’s correlations with the BPI and PROMIS scales further support its convergent validity. The ABCs offers a nuanced and functional approach to evaluating pain with evidence for its validity in chronic pain patients.

## Figures and Tables

**Table 1:** Participant Characteristics.

Characteristic	Cohort 1 (N = 297)	Cohort 2 (N = 304)	Retest participants (N = 194)
Age^a^	34 [28-43]	38 [30-51]	40 [32-52]
Sex^b^			
Female	164 (55%)	144 (48%)	95 (51%)
Male	132 (44%)	152 (51%)	90 (49%)
Prefer not to say	1 (0.3%)	2 (0.7%)	0 (0%)
Race^b^			
Asian	18 (6.1%)	10 (3.4%)	6 (3.2%)
African American/Black	26 (8.8%)	19 (6.4%)	11 (5.9%)
White	210 (70.7%)	238 (80%)	148 (80%)
Mixed	29 (9.8%)	21 (7.0%)	13 (7.0%)
Other	13 (4.4%)	10 (3.4%)	7 (3.8%)
Pain duration^c^			
6-12 mo	-	22 (7.4%)	10 (5.4%)
1-2 y	-	16 (5.4%)	9 (4.9%)
2-5 y	-	76 (26%)	47 (25%)
5-10 y	-	106 (36%)	68 (37%)
10-20 y	-	78 (26%)	51 (28%)

a Median [interquartile range].

b n (%).

c Mean (standard deviation).

**Table 2:** Final Confirmatory Factor Analysis of the Activity-Based Checks of Pain Scales.

CFI	TLI	RMSEA	SRMR
0.92	0.91	0.09	0.05

CFI = comparative fit index; TLI = Tucker-Lewis index; RMSEA = root meanerror approximation; SRMR = standardized root mean-square residual.

**Table 3:** Descriptive Statistics and Internal Consistency Reliability for the Activity-Based Checks of Pain Subscales.

Subscale	N	Mean	Standard Deviation	Range	Cronbach’s Alpha	Omega
Complex multiplanar	303	3.49	2.14	0.0-9.2	0.87	0.87
Sitting/hip flexor	301	2.68	2.12	0.0-9.0	0.90	0.90
Standing/ambulation	301	4.04	2.42	0.0-9.5	0.94	0.94
Lightweight unilateral	298	1.28	1.49	0.0-8.5	0.84	0.84

**Table 4:** Activity-Based Checks of Pain Subscale Correlations with BPI and PROMIS-29.

	Activity-Based Checks of Pain Subscales				
	Complex Multiplanar	Sitting/Hip Flexor	Standing/Ambulation	Lightweight Unilateral
BPI (pain interference)	0.58	0.51	0.59	0.48
PROMIS-29 (pain interference)	0.70	0.52	0.60	0.49

Note: All values were significant (*p* < .001).

BPI = Brief Pain Inventory; PROMIS = Patient-Reported Outcomes Measurement Information Systems.

**Table 5:** Spearman’s Correlation Coefficients for Analogous Variables.

ABCs versus PROMIS Items	Rho
ABC walking pain vs. BPI walking ability	0.66
ABC walking pain vs. PROMIS “Are you able to walk at a normal speed?”	−0.73
ABC prolonged standing vs. PROMIS “How much did pain prevent you from standing for more than 30 minutes?”	0.68
ABC prolonged standing vs. PROMIS “How much did pain prevent you from sitting for more than 30 minutes?”	0.63
ABC getting out of bed vs. PROMIS “Are you able to sit down in and stand up from a low, soft couch?”	−0.57
ABC getting out of a chair vs. PROMIS “Are you able to stand from an armless straight chair?”	−0.59
ABC grocery shopping vs. PROMIS “How much did pain interfere with doing your tasks away from home (e.g., getting groceries, running errands)?”	0.62
ABC housework vs. BPI housework	0.49
ABC housework vs. PROMIS “How much did pain interfere with work around the home?”	0.65
ABC picking up 20 pounds vs. PROMIS “Are you able to carry a heavy object (over 10 pounds/5 kg)?”	−0.63
ABC eating vs. PROMIS “Are you able to cut your food using eating utensils?”	−0.23
ABC opening a bottle of medicine vs. PROMIS “Are you able to open a tight or new jar?”	−0.54
ABC walking upstairs vs. PROMIS “Are you able to go up and down stairs at a normal pace?”	−0.71
ABC personal care/grooming vs. PROMIS “Are you able to shave your face or apply makeup?”	−0.44
ABC sitting up vs. PROMIS “Are you able to sit on the edge of a bed?”	−0.45
ABC laundry vs. PROMIS “How much did pain interfere with your household chores?”	0.61

ABC = Activity-Based Checks of Pain; BPI = Brief Pain Inventory; PROMIS = Patient-Reported Outcomes Measurement Information Systems.
